# Multi-omics triangulation identifies complement factor H as a genetically supported protective factor in IgA nephropathy

**DOI:** 10.1093/ckj/sfag176

**Published:** 2026-05-29

**Authors:** Ningjun Shao, Kuibi Tan, Ping Chen, Qun Luo

**Affiliations:** Department of Nephrology, Ningbo No.2 Hospital, Wenzhou Medical University, Ningbo, Zhejiang Province, China; Department of Nephrology, Ningbo No.2 Hospital, Wenzhou Medical University, Ningbo, Zhejiang Province, China; Department of Nephrology, Ningbo No.2 Hospital, Wenzhou Medical University, Ningbo, Zhejiang Province, China; Department of Nephrology, Ningbo No.2 Hospital, Wenzhou Medical University, Ningbo, Zhejiang Province, China

**Keywords:** alternative pathway, complement factor H, IgA nephropathy, Mendelian randomization, multi-omics

## Abstract

**Background:**

IgA nephropathy is the most prevalent primary glomerulonephritis globally. Dysregulation of the complement system, specifically the alternative pathway, is a critical driver of its pathogenesis. While genome-wide association studies consistently map a primary susceptibility locus to the complement factor H gene cluster, extensive linkage disequilibrium and complex structural variations have historically obscured precise causal targets.

**Methods:**

We employed a multi-omics triangulation framework to evaluate the genetic association and potential protective effects of circulating complement proteins in IgA nephropathy. The analytical pipeline integrated large-scale plasma proteomics, blood-derived expression quantitative trait loci, Bayesian colocalization, computational clinical transcriptomics, and phenome-wide association studies. Two-sample, bidirectional, and multivariable Mendelian randomization analyses were performed using summary-level data from global genome-wide association studies.

**Results:**

Genetically predicted higher circulating levels of complement factor H were significantly associated with a lower risk of IgA nephropathy. Bayesian colocalization decoupled the complex locus, isolating this protective signal to a single missense variant within the complement factor H gene and demonstrating independence from the adjacent complement factor H-related 1 gene. Spatial profiling and clinical transcriptomics suggested a dual-compartment model: the liver supplies the systemic pool, while intrinsic renal cells mount a hypothesized severity-driven compensatory upregulation of complement factor H during active disease. Furthermore, phenome-wide association studies indicated that enhancing complement factor H offers dual protective benefits against IgA nephropathy and age-related macular degeneration without broad chronic systemic pleiotropy.

**Conclusions:**

This study provides convergent genomic and transcriptomic evidence supporting complement factor H as a genetically supported protective factor in IgA nephropathy. Genetic variation consistent with enhanced complement factor H-mediated regulation is associated with reduced disease susceptibility. These findings provide a theoretical framework for exploring direct recombinant supplementation therapies and offer genetic support for the ongoing clinical development of upstream alternative pathway inhibitors.

KEY LEARNING POINTS **What was known:**

- Dysregulation of the alternative complement pathway is a critical driver of IgA nephropathy pathogenesis.
- Genome-wide association studies consistently map a major susceptibility locus to the 1q32 gene cluster, which encodes complement factor H (CFH) and its related paralogs.
- Extensive linkage disequilibrium and complex structural variations within this cluster have obscured precise causal variants, complicating efforts to identify exact therapeutic targets.

**This study adds:**

- Multi-omics triangulation and Bayesian colocalization successfully decoupled the 1q32 locus, confirming that CFH is a genetically supported protective factor in IgA nephropathy, independent of CFHR1.
- Clinical transcriptomics support a hypothesized severity-driven compensatory upregulation of intra-renal CFH during active disease, highlighting a dual-compartment physiological defense mechanism.
- Phenome-wide association screening demonstrates the chronic systemic safety of CFH enhancement and reveals a dual protective benefit against age-related macular degeneration.

**Potential impact:**

- The genomic evidence for CFH is consistent with the rationale underlying the clinical development of upstream alternative pathway inhibitors, such as iptacopan, for treating IgA nephropathy.
- These findings serve as a hypothesis-generating observation for exploring direct recombinant CFH supplementation (e.g. GEM103) as a novel precision therapeutic candidate, requiring rigorous preclinical validation.
- The identified shared protective effects suggest that modulating the CFH pathway could offer comprehensive clinical benefits for patients with concurrent complement-mediated renal and ocular conditions.

## BACKGROUND

IgA nephropathy (IgAN) remains the most prevalent primary glomerulonephritis globally, causing a substantial proportion of patients to progress to end-stage renal disease [1]. Its pathogenesis is widely described as a “multi-hit” cascade, culminating in the mesangial deposition of nephritogenic immune complexes [2]. While optimized supportive care can temporarily reduce proteinuria, it largely fails to arrest the underlying immunological drive [3]. Recently, the complement system, specifically the dysregulated overactivation of the alternative pathway (AP), has emerged as a critical driver of glomerular inflammation, podocyte effacement, and progressive fibrotic scarring [4].

Deciphering the genetic architecture of AP dysregulation has been a major focus of large-scale genome-wide association studies (GWAS). These studies consistently map a primary susceptibility locus to chromosome 1q32 [5, 6]. This region encodes Complement Factor H (CFH), the primary negative regulator of the AP, alongside its related paralogs (CFHR1–5). However, translating this broad genetic signal into a precise therapeutic target remains challenging. The 1q32 cluster is characterized by extensive linkage disequilibrium (LD) and complex structural variations [7]. This structural complexity significantly hinders the identification of definitive causal variants. Furthermore, conventional associations between circulating complement levels and disease severity are frequently confounded by reverse causality, thereby limiting valid causal inference. For instance, acute nephritic flares often cause non-specific urinary loss or reactive hepatic synthesis of complement proteins [8]. Consequently, it remains uncertain which specific elements within the 1q32 cluster drive disease susceptibility.

Resolving this locus complexity is a clinical priority, particularly as a new generation of proximal AP inhibitors advances in clinical trials [9]. To safely and effectively guide these complement-directed therapeutics, the underlying targets require genomic validation. In this study, we used a multi-omics triangulation strategy to bypass the confounding biases of observational research [10, 11]. By integrating large-scale plasma proteomics, blood-derived expression quantitative trait loci (eQTL), Bayesian colocalization, and *in silico* clinical transcriptomics, we sought to evaluate the genetic association of the CFH-CFHR locus with disease risk. Through this orthogonal genetic framework, paired with phenome-wide association studies (PheWAS), we aim to provide clearer insights into the 1q32 locus and offer hypothesis-generating genomic evidence to inform the ongoing clinical development of complement-directed IgAN therapeutics.

## MATERIALS AND METHODS

### Study design and data sources

We used a multi-omics triangulation framework to evaluate the genetic association and potential protective effects of circulating complement proteins in IgAN. The analytical pipeline included two-sample Mendelian randomization (MR), bidirectional MR, multivariable MR (MVMR), Bayesian colocalization, eQTL analysis, *in silico* clinical transcriptomics, and PheWAS. To maximize target coverage, genetic instruments proxying the circulating levels of candidate complement proteins were obtained from two large-scale plasma proteomics GWAS. Summary statistics for CFH, complement component 3 (C3), and A proliferation-inducing ligand (APRIL) were sourced from the UK Biobank Pharma Proteomics Project (UKB-PPP), which quantified plasma proteins using the Olink Proximity Extension Assay [12]. Data for CFH-related 1 (CFHR1) were extracted from the deCODE Genetics proteomics GWAS using the SomaScan multiplexed aptamer platform [13]. For the disease outcome, summary-level data were extracted from the largest available global IgAN GWAS meta-analysis [6]. Both exposure and outcome datasets were restricted to individuals of European ancestry to reduce population stratification bias. No ethical approval or informed consent was required because this study used publicly available, de-identified summary-level data. Circulating complement regulators were prioritized in this analytical framework based on the availability of robust plasma pQTL data. Consequently, membrane-bound complement activation regulators within the 1q32 locus, such as membrane cofactor protein (MCP/CD46), were excluded from the baseline MR pipeline due to their lack of physiological secretion into the systemic circulation and the subsequent absence of valid circulating protein genetic instruments in public summary statistics.

### Instrumental variable selection and forward Mendelian randomization

Instrumental variables (IVs) for the forward MR analysis were selected based on the three core MR assumptions. We identified single nucleotide polymorphisms that achieved genome-wide significance (*P* < 5 × 10⁻⁸) and subsequently clumped them for LD (*r*² < 0.001, clumping window >10 000 kb) against the 1000 Genomes Project European reference panel. To prevent weak instrument bias, we evaluated instrument strength using the *F*-statistic (*F* = β²/SE²), excluding variants with *F* < 10. Primary effect estimates were computed using the inverse variance weighted (IVW) method with multiplicative random effects. Potential horizontal pleiotropy was addressed through sensitivity analyses using weighted median and MR-Egger regression models. Finally, we evaluated instrumental heterogeneity using Cochran’s Q statistic and tested for directional horizontal pleiotropy via the MR-Egger intercept term.

### Bidirectional Mendelian randomization

To assess reverse causation (the possibility that systemic alteration of target proteins is a secondary consequence of IgAN progression or renal leakage), we conducted a bidirectional MR analysis. Genetically predicted susceptibility to IgAN was used as the exposure. Independent genome-wide significant IVs for IgAN (*P* < 5 × 10⁻⁸, *r*² < 0.001) were extracted and mapped to the full genome summary statistics of the identified target proteins. Reverse genetic estimates were computed using the IVW method, complemented by MR-Egger and weighted median analyses.

### Multivariable MR at the 1q32 locus

Given the complex structural variations and extreme LD within the CFH-CFHR gene cluster at chromosome 1q32, we initially attempted an MVMR approach to disentangle the independent effects of CFH and CFHR1. IVs associated with either CFH or CFHR1 (*P* < 1 × 10^-4^) were pooled and clumped to form a combined instrument matrix. We applied the MVMR-IVW model. To formally quantify the multicollinearity resulting from genetic correlation at this locus, we calculated variance inflation factors (VIF) and phenotypic matrix condition numbers.

### Bayesian colocalization analysis

To assess whether the implicated proteins share the same underlying genetic signals with IgAN risk, independent of LD confounding, we performed Bayesian colocalization using the coloc R package (version 5.2). Summary statistics from the IgAN GWAS and the corresponding *cis-*protein quantitative trait loci (*cis-*pQTL) regions (defined as ±1 Mb around the top protein-associated variant) were extracted. Genomic coordinates were harmonized to the GRCh38 reference panel, and effect alleles were aligned. The prior probabilities that a variant is associated with trait 1 (p1), trait 2 (p2), or both traits (p12) were set at 1 × 10^-4^, 1 × 10^-4^, and 1 × 10⁻⁵, respectively. A posterior probability of hypothesis 4 (PP.H4) > 0.80 was considered strong evidence for colocalization (a shared variant).

### Transcriptome-wide Mendelian randomization (eQTL analysis)

To extend our systemic genetic findings to the transcriptional level, we performed an eQTL-based MR. Independent *cis-*eQTL instruments (*P* < 5 × 10⁻⁸) regulating the whole-blood mRNA expression of CFH were extracted from the eQTLGen Consortium dataset [14]. Because these instruments are derived from blood, this analysis estimates the effect of systemic transcriptional regulation rather than kidney-specific local expression. Due to the limited number of available instruments, the pleiotropy-robust weighted median model was prioritized for analysis.

### *In silico* clinical transcriptomics and functional co-expression networks

To relate the systemic genetic findings to the local disease microenvironment and explore the clinical dynamics of CFH transcription, we used independent clinical transcriptomic datasets. Differential intra-renal CFH mRNA expression was analyzed using microdissected glomerular microarray data from the GSE93798 cohort, comparing patients with IgAN to healthy living donors. To correlate CFH expression with clinical severity indices (such as estimated glomerular filtration rate, proteinuria, and serum creatinine), multi-center cohort data were analyzed using the Nephroseq v5 platform. To investigate the functional context of local CFH upregulation and explore the hypothesized compensatory defense mechanism, we conducted a co-expression network analysis within the IgAN microenvironment. Genes exhibiting the strongest positive correlation with CFH expression (Pearson’s r) were extracted and subjected to Gene Ontology (GO) biological process enrichment analysis via Metascape and DAVID to distinguish homeostatic regulatory networks from pro-inflammatory pathways.

### Bulk and single-cell transcriptomic profiling

Bulk RNA-sequencing consensus expression data (measured in normalized transcripts per million, nTPM) across major human tissues were retrieved from the Human Protein Atlas (HPA) to assess systemic tissue specificity [15]. To examine the localized expression architecture within the human kidney microenvironment, we used the CZ CELLxGENE Discover platform [16]. Curated single-cell RNA-sequencing (scRNA-seq) datasets of healthy adult human kidneys were queried to extract the relative expression levels of CFH across distinct renal cell populations, including mesangial cells, glomerular endothelial cells, and podocytes.

### PheWAS

To screen for potential pleiotropic associations and estimate the genetic liability for off-target effects associated with CFH modulation, we conducted a PheWAS. The ieugwasr R package was used to query the MRC IEU OpenGWAS database [17], using the lead colocalized variant for CFH (rs6677604) as the genetic proxy. A significance threshold of *P* < 5 × 10⁻⁵ was applied to minimize false positives. Non-disease traits, such as technical measurements, were excluded to focus on clinically relevant biological endpoints. Although PheWAS assesses linear genetic tendencies, it does not replace preclinical toxicological evaluations for systemic complement inhibition.

### Statistical analysis

All statistical analyses were conducted using R software (version 4.5.1). MR analyses were performed using the TwoSampleMR package. Colocalization and MVMR analyses were performed using the coloc and MVMR packages, respectively. Data visualizations were generated using ggplot2 and patchwork. All reported *P*-values are two-sided, and statistical significance was defined as *P* < 0.05 unless otherwise specified.

## RESULTS

### Identification of circulating complement proteins associated with IgAN risk

**Figure 1:**
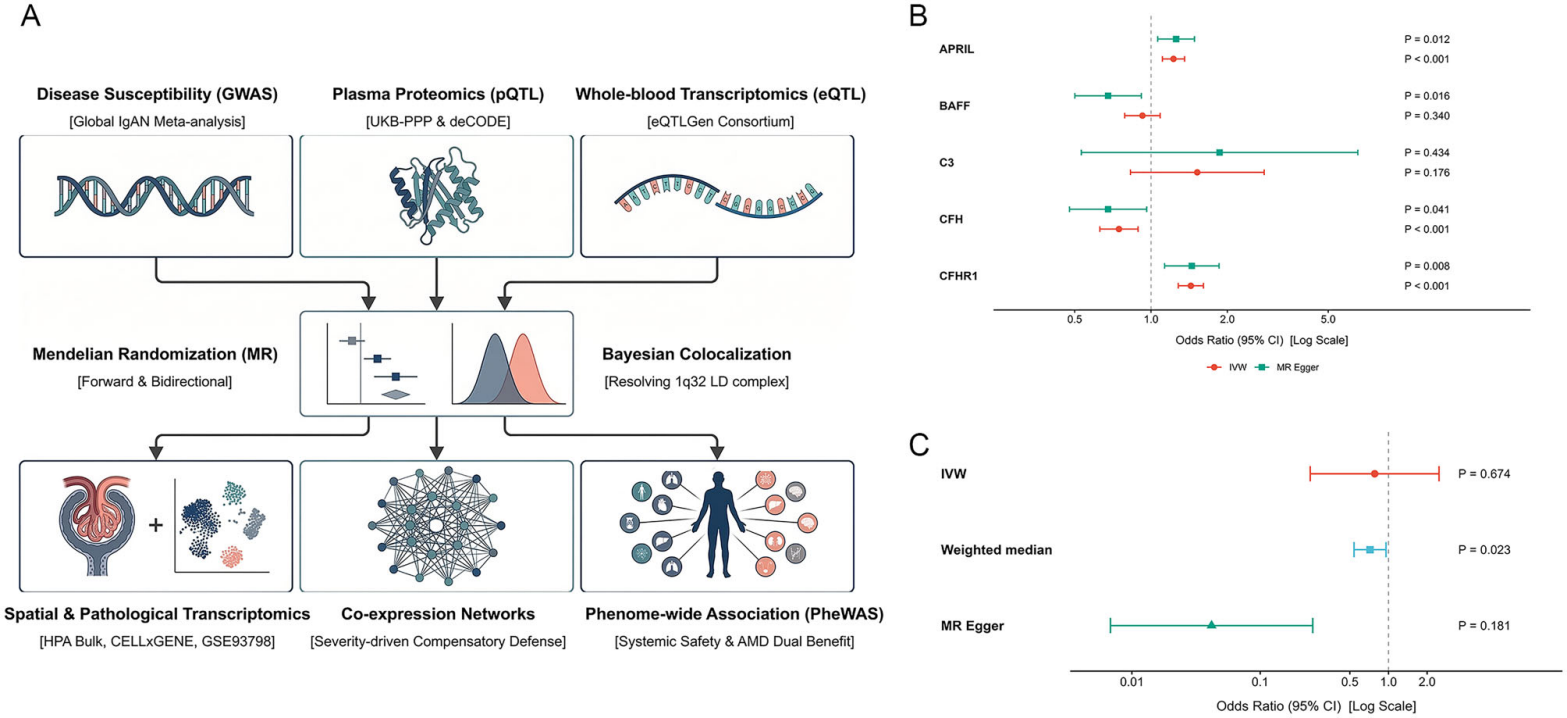
Study design and Mendelian randomization analysis for complement proteins in IgA nephropathy. (A) Overview of the multi-omics triangulation framework. The analytical pipeline integrates two-sample Mendelian randomization (MR), Bayesian colocalization, expression quantitative trait loci (eQTL) MR, and transcriptomic profiling. (B) Forward MR analysis estimating the causal effects of genetically predicted circulating complement protein levels on IgA nephropathy (IgAN) risk. (C) Transcriptome-wide MR (eQTL) analysis evaluating the effect of whole-blood CFH mRNA expression on IgAN risk, applying the weighted median model to address instrumental heterogeneity.

The overall multi-omics triangulation study design, aimed at systematically evaluating the genetic association and potential protective effects of circulating proteins in IgAN pathogenesis, is outlined in Fig. 1A. Using a two-sample MR framework, our primary IVW analysis indicated that genetically predicted circulating levels of CFH were significantly associated with a reduced risk of IgAN [odds ratio (OR) = 0.747, 95% confidence interval (CI): 0.628–0.889, *P* < .001; Fig. 1B, Supplementary Table S1]. The selected genetic instruments for the candidate proteins showed adequate F-statistics, minimizing the potential for weak instrument bias (Supplementary Table S2). The protective trend of CFH was consistent across pleiotropy-robust methods, including the weighted median (OR = 0.742, *P* = .018) and MR-Egger (OR = 0.677, *P* = .041) models.

Our initial analysis also indicated that genetically predicted elevations in CFHR1 and APRIL were associated with increased IgAN risk (CFHR1: IVW OR = 1.436, *P* < .001; APRIL: IVW OR = 1.231, *P* < .001). C3 showed a nominally significant risk association under the weighted median model (OR = 1.651, *P* = .034). Sensitivity analyses, including the MR-Egger intercept test and Cochran’s Q statistic, did not detect severe directional horizontal pleiotropy across these targets (Supplementary Table S3).

### Bidirectional MR supports a unidirectional genetically predicted trajectory for CFH

A challenge in genetic epidemiology is differentiating upstream causal drivers from secondary disease biomarkers. To address this, we performed bidirectional MR using 25 independent genome-wide significant variants for IgAN to evaluate their effect on circulating target proteins (Supplementary Table S4). Genetically predicted IgAN yielded no significant genetic effect on circulating CFH levels across analytical models (IVW Beta = −0.154, *P* = .152; MR-Egger *P* = .067; weighted median *P* = .635). Similar non-significant reverse effects were observed for other components, including C3 (IVW *P* = .698) and CFHR1 (IVW *P* = .067). These data support a unidirectional association, consistent with the possibility that genetic variation in CFH operates upstream of disease processes rather than reflecting a secondary consequence.

### Whole-blood eQTL MR links systemic transcriptional regulation to disease risk

To extend our systemic baseline analysis to the transcriptional level, we used independent eQTL instruments derived from whole blood (eQTLGen Consortium). Under this framework, genetically predicted upregulation of blood CFH mRNA demonstrated a protective association against IgAN (OR = 0.718, 95% CI: 0.539–0.955, *P* = .023; Fig. 1C, Supplementary Table S5). Significant instrumental heterogeneity was detected (Cochran’s Q *P* < .001; Supplementary Table S6). This necessitated the use of the pleiotropy-robust weighted median method, while the MR-Egger intercept confirmed the absence of directional pleiotropy (*P* = .189). These blood-derived eQTL data support the systemic genetic evidence, consistent with the concept that enhanced systemic transcriptional regulation of CFH confers disease protection.

### Colocalization analysis decodes 1q32 locus complexity and highlights CFH

While MR uses genetic instruments to infer potential causal relationships, it can be confounded by localized LD. Given the extensive LD at the 1q32 complement locus, we initially attempted a MVMR framework to mutually adjust for the effects of CFH and CFHR1. The MVMR model showed severe multicollinearity, indicated by highly inflated VIF, rendering independent effect estimations mathematically unresolvable (both adjusted *P* > .05; Supplementary Table S7).

**Figure 2:**
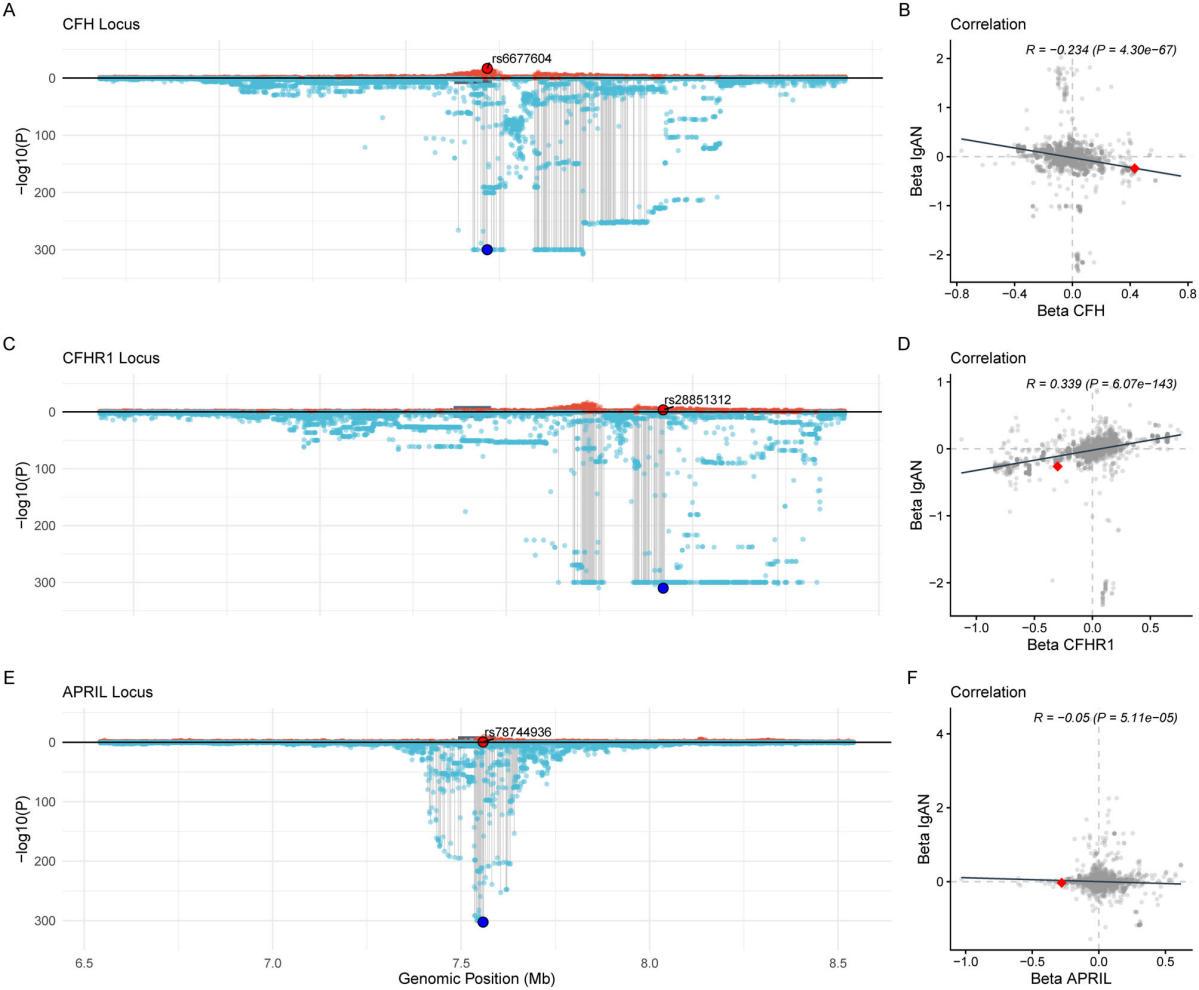
Bayesian colocalization analysis at the 1q32 locus and APRIL locus. (A, C, E) Regional association plots comparing the genetic association signatures for circulating protein levels (upper panels) and IgAN risk (lower panels). (A) Colocalization results for CFH and IgAN, indicating the posterior probability of hypothesis 4 (PP.H4) and the peak missense variant rs6677604. (B) Scatter plot of the harmonized genetic effect sizes (Pearson correlation, r) for variants within the CFH locus. (C, D) Colocalization results for CFHR1 and IgAN, displaying PP.H4 and the posterior probability of hypothesis 3 (PP.H3). (E, F) Colocalization results between circulating APRIL levels and IgAN risk.

This statistical limitation necessitated the use of Bayesian colocalization. The analysis provided evidence that circulating CFH levels and IgAN risk share a single genetic signal at the 1q31.3 locus (PP.H4 = 98.66%; Fig. 2A). The shared signal peaked at rs6677604, a functional missense variant within the CFH gene. Comparison of the harmonized genetic effect sizes revealed a significant inverse correlation (Pearson *r* = −0.234, *P* = 4.30 × 10^-67^; Fig. 2B), indicating that variants predicting higher circulating CFH protein levels correspond to a decreased risk of IgAN.

Despite initial univariable MR analyses suggesting an association for CFHR1, colocalization yielded no evidence for a shared genetic signal between CFHR1 and IgAN (PP.H4 < 1 × 10⁻⁶). This is consistent with two distinct genetic signals (PP.H3 = 99.99%; Fig. 2C, D). This suggests that the nominal MR signal for CFHR1 in our dataset is likely an artifact driven by extensive LD with the adjacent CFH locus, supporting CFH as the most likely genomic driver within this locus. Additionally, colocalization did not find strong evidence for a shared *cis-*pQTL driving both circulating APRIL levels and IgAN risk (PP.H4 = 1.28%; Fig. 2E, F).

### Expression profiling suggests a liver-kidney dual-compartment distribution

**Figure 3:**
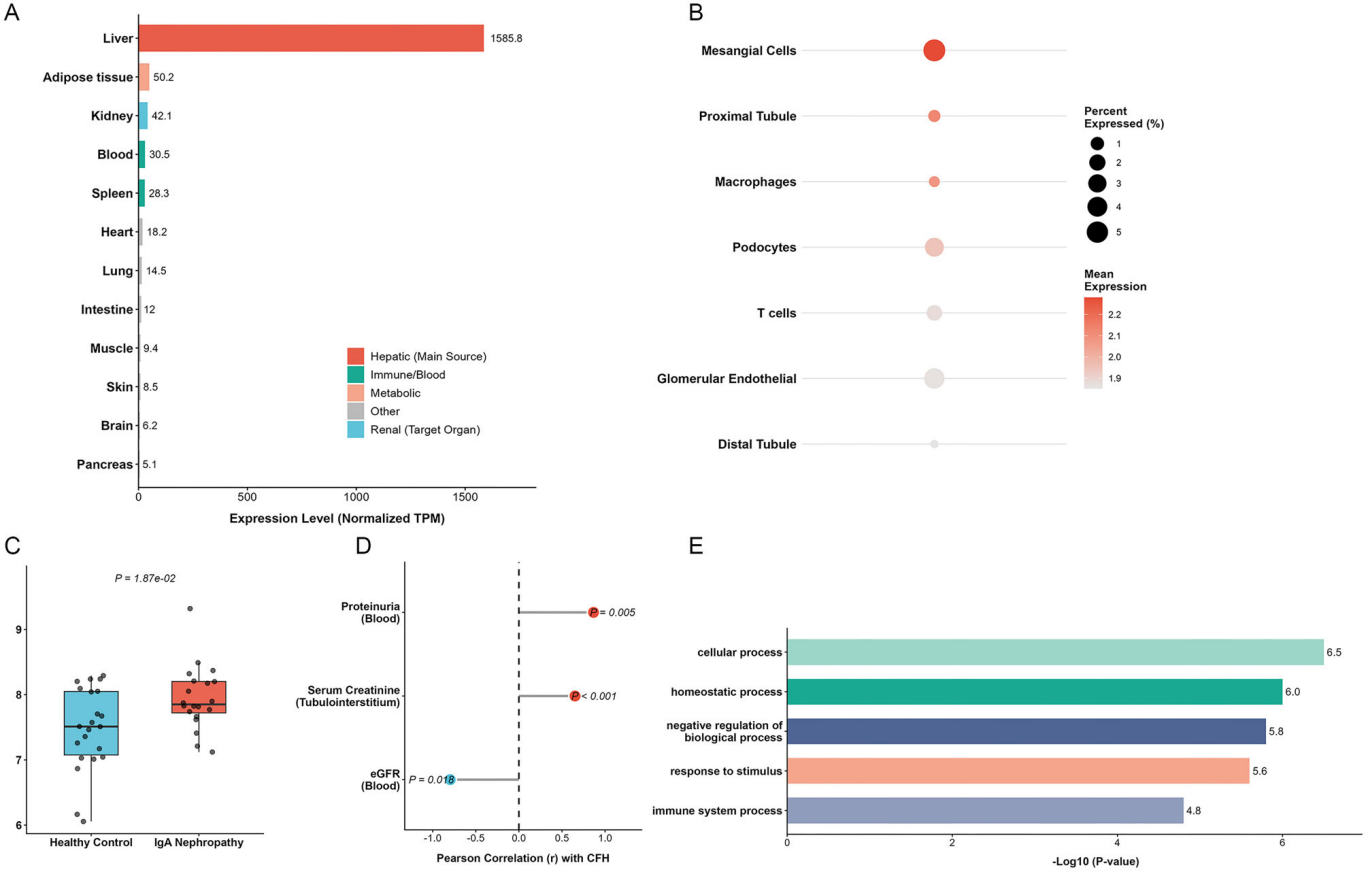
Spatial profiling and clinical transcriptomics of CFH. (A) Systemic tissue specificity of CFH mRNA expression using Human Protein Atlas (HPA) bulk RNA-seq data. (B) Single-cell RNA-sequencing (scRNA-seq) map of the human kidney microenvironment (CZ CELLxGENE Discover platform), displaying CFH expression across cell types including mesangial and glomerular endothelial cells. (C) Differential intra-renal CFH mRNA expression in microdissected glomeruli of patients with IgAN compared to healthy controls (GSE93798 cohort, Wilcoxon test). (D) Clinical correlations derived from the Nephroseq platform. The plot shows Pearson correlation coefficients (r) between CFH expression and clinical indices (serum creatinine, proteinuria, and estimated glomerular filtration rate). (E) Gene Ontology (GO) enrichment analysis of the CFH co-expression network within the IgAN microenvironment.

Given the genetic evidence supporting CFH, we sought to determine its physiological distribution in healthy tissues. Bulk RNA-seq analysis confirmed that the liver is the predominant systemic source of CFH mRNA (nTPM = 1585.8). This drives the circulating plasma pool with expression levels substantially higher than those in other organs (Fig. 3A). To examine the local expression architecture within the healthy renal microenvironment, we analyzed standardized scRNA-seq datasets. Within the kidney, distinct local expression signatures were observed in mesangial cells, glomerular endothelial cells, and resident macrophages (Fig. 3B). This distribution pattern leads us to hypothesize a dual-compartment physiological model: the liver supplies the bulk circulating pool, while intrinsic glomerular cells may provide basal on-site regulation.

### Hypothesized severity-driven compensatory CFH transcriptional network in the local IgAN microenvironment

To investigate how the local microenvironment responds during active pathogenesis, we conducted computational evaluations using independent transcriptomic cohorts. Analysis of microdissected glomeruli (GSE93798) revealed a significant upregulation of intra-renal CFH mRNA in patients with IgAN compared to healthy controls (median Log2 expression 7.85 vs. 7.51, *P* = .019; Fig. 3C). We further evaluated multi-cohort clinical correlations via the Nephroseq platform. The upregulation of CFH mRNA was positively correlated with disease severity indices. In the tubulointerstitium, CFH expression showed a strong positive correlation with serum creatinine levels (*r* = 0.651, *P* < .001). In the systemic circulation, CFH transcript levels were positively correlated with proteinuria (*r* = 0.866, *P* = .005) and inversely correlated with estimated glomerular filtration rate (eGFR) (*r* = −0.797, *P* = .018) (Fig. 3D). Given the genetically protective baseline of CFH, we hypothesize this could reflect a severity-driven compensatory defense model: as complement-mediated injury exacerbates (accompanied by massive local consumption and urinary loss of the CFH protein [18]), both local and systemic compartments mount a reactive transcriptional surge to counteract ongoing alternative pathway activation.

GO enrichment analysis of the CFH co-expression network within the IgAN microenvironment demonstrated that this highly synchronized gene cluster was predominantly enriched in the “homeostatic process” (−Log10 *P* = 6.0) and “negative regulation of biological process” (−Log10 *P* = 5.8) (Fig. 3E). While associative in nature, the functional network supports the interpretation that local CFH upregulation reflects a compensatory homeostatic response rather than a pro-inflammatory mechanism.

### PheWAS screening for potential pleiotropic associations

**Figure 4:**
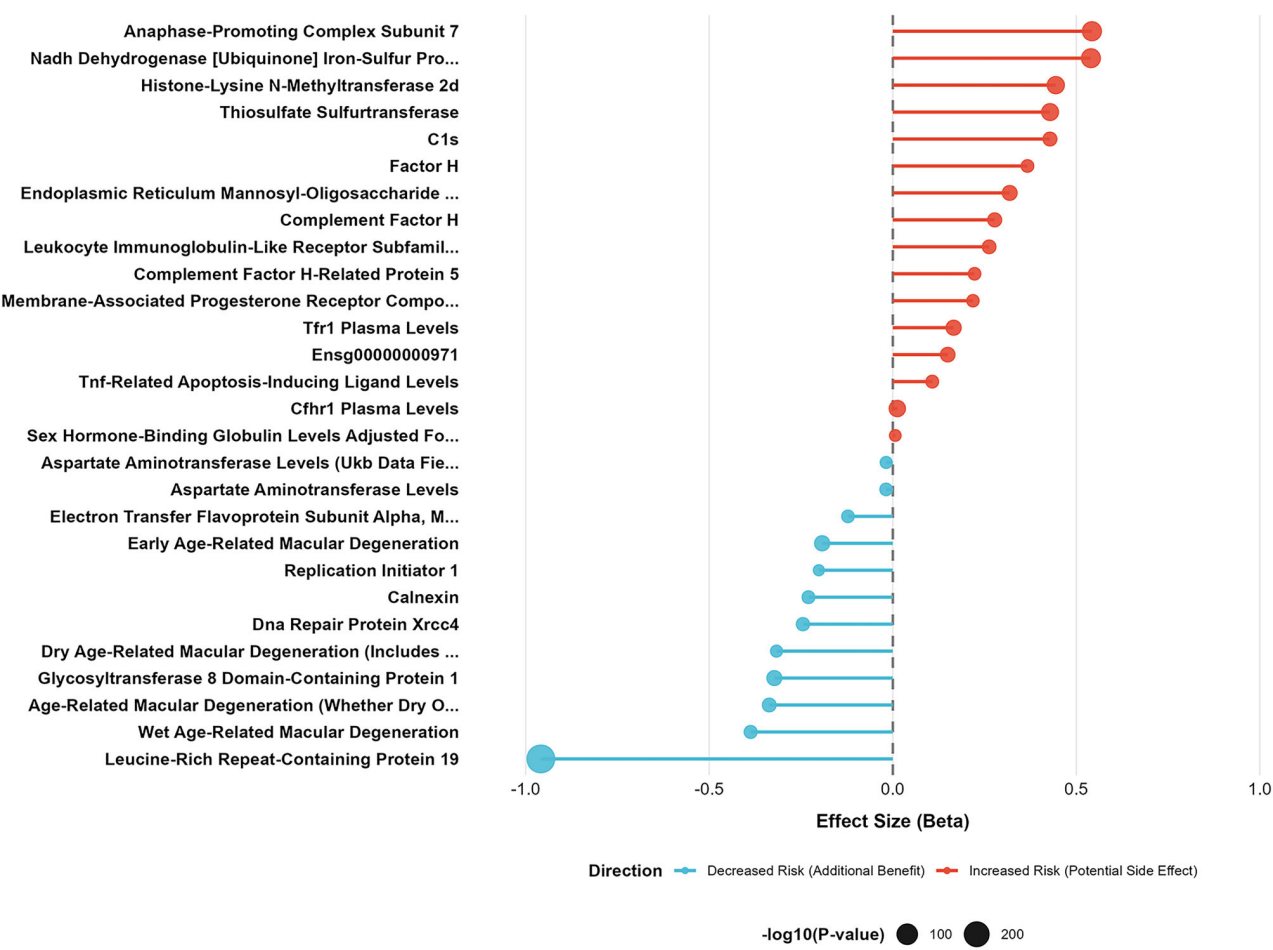
We agree with this recommendation to enhance accessibility for visually impaired readers. The explicit references to color have been removed, and the caption has been updated to describe the directional orientation of the data instead. Please replace the existing caption with the following updated text: "Phenome-wide association study (PheWAS) of the lead CFH variant. The plot illustrates cross-phenotype associations for the colocalized CFH variant (rs6677604) across human traits and diseases using the MRC IEU OpenGWAS database. Only traits reaching the significance threshold (P < 5 × 10⁻⁵) are displayed. Effect sizes (beta) are harmonized to the CFH-increasing allele, where positive effect sizes indicate an increased risk or elevated biomarker levels associated with the allele, and negative effect sizes denote a decreased risk.”.

To evaluate the broader phenotypic impact and estimate the genetic liability for off-target effects associated with CFH modulation, we conducted a PheWAS using the lead colocalized variant (rs6677604) (Fig. 4). At a stringent significance threshold (*P* < 5 × 10⁻⁵), the CFH-increasing allele was significantly associated with alterations in the circulating levels of several other local proteins (e.g. CFHR5), reflecting the complex *cis-*regulatory landscape of the 1q32 locus.

Clinically, the most prominent cross-phenotype associations were observed exclusively in ophthalmological traits. Genetically predicted upregulation of CFH was strongly associated with a decreased risk of age-related macular degeneration (AMD), encompassing both early and late-stage subtypes (e.g. dry AMD: beta = −0.317, *P* = 7.29 × 10⁻¹²; wet AMD: beta = −0.428, *P* = 3.63 × 10⁻¹²). This is consistent with the established paradigm that complement alternative pathway dysregulation is a central driver of AMD pathogenesis. Consequently, therapeutic strategies aiming to enhance CFH function could theoretically offer dual protective benefits across renal and ocular compartments.

No extensive off-target pleiotropic diseases (such as systemic autoimmune, cardiovascular, or metabolic disorders) crossed the significance threshold. Although this screening indicates an absence of broad chronic pleiotropy, a key limitation of genetic epidemiology is that a lifelong genetic baseline cannot effectively simulate acute procedural or pharmacological risks. For instance, systemic alternative pathway inhibition is historically associated with an increased susceptibility to encapsulated bacterial infections, notably *Neisseria meningitidis*. Consequently, while these PheWAS findings support the long-term systemic safety of CFH enhancement, they do not circumvent the need for rigorous preclinical toxicological evaluations and strict vaccination protocols in clinical trials.

### Translational implications for alternative complement pathway modulation

These genomic and transcriptomic insights provide a framework to evaluate the current pharmacological pipeline targeting the alternative complement pathway (Table 1). Upstream inhibition of components such as factor B or factor D is designed to replicate the alternative pathway-suppressive effects of CFH, and selective factor B inhibitors like iptacopan are advancing in clinical trials for IgA nephropathy. Furthermore, our genetic data offer a hypothesis-generating observation regarding direct recombinant CFH supplementation, such as GEM103. Accordingly, this should not be considered clinically actionable but instead a theoretical repurposing candidate that necessitates rigorous preclinical *in vivo* validation prior to clinical translation.

**Table 1:** Pharmacological pipeline targeting the alternative complement pathway in IgA nephropathy.

Drug candidate	Pharmaceutical company	Molecular target	Complement pathway	Mechanism of Action (MoA) & relationship to CFH	Current clinical phase in IgAN
Iptacopan (LNP023)	Novartis	Complement Factor B (CFB)	Alternative	Reversible CFB inhibitor. Mimics CFH by preventing the formation of the alternative C3 convertase (C3bBb).	Phase III (APPLAUSE-IgAN)
Pegcetacoplan (APL-2)	Apellis	C3 and C3b	Alternative & common	PEGylated peptide binding C3. Functions downstream of CFH, preventing C3 cleavage and broad complement amplification.	Phase II/III
Vemircopan (ALXN2050)	AstraZeneca/Alexion	Complement Factor D (CFD)	Alternative	CFD inhibitor. Mimics CFH by blocking the cleavage of CFB, thus halting the alternative pathway amplification loop.	Phase II
Cemdisiran	Alnylam	C5 (Hepatic synthesis)	Terminal	RNAi therapeutic silencing liver C5 production. Blocks terminal MAC formation downstream of alternative pathway activation.	Phase II
Zilucoplan	UCB	C5	Terminal	Macrocyclic peptide C5 inhibitor. Prevents terminal C5b-9 (MAC) formation, mitigating downstream tissue injury.	Phase II
Narsoplimab (OMS721)	Omeros	MASP-2	Lectin	MASP-2 monoclonal antibody. Targets the lectin pathway, which often co-activates with the alternative pathway in IgAN.	Phase III (ARTEMIS-IGAN)
GT009/GEM103 *	Gemini therapeutics	Recombinant CFH (rCFH)	Alternative	Full-length recombinant human CFH. Direct target restoration. Currently evaluated in AMD, showing potential for systemic administration in renal diseases.	Preclinical/repurposing candidate

* Investigated primarily for age-related macular degeneration (AMD); represents a theoretical drug repurposing candidate that necessitates rigorous preclinical in vivo validation prior to clinical translation in IgA nephropathy.

## DISCUSSION

The genetic architecture of IgAN is characterized by complement system dysregulation. However, translating broad GWAS risk loci into actionable pharmacological targets remains a challenge [1]. By integrating proteomics, whole-blood transcriptomics, single-cell profiling, and *in silico* clinical validation within a MR framework, the present study identifies circulating CFH as a genetically supported protective factor in IgAN pathogenesis. We provide genomic evidence supporting a model in which genetic upregulation of the CFH-mediated AP brake is associated with reduced disease susceptibility.

A methodological requirement of this study was decoupling the highly complex CFH-CFHR gene cluster at chromosome 1q32. Observational and early genetic studies have consistently implicated this locus, but extensive LD and structural variations have historically obscured the precise causal variants [1]. Our MVMR approach mathematically failed to separate these paralogs due to severe multicollinearity. By deploying Bayesian colocalization, we show that the IgAN protective signal colocalizes with CFH at the single-nucleotide level (rs6677604).

We recognize the residual confounding inherent to regions of high LD. The peak variant, rs6677604, represents a locus-level association that likely captures a composite haplotypic effect across the CFH-CFHR cluster rather than a singular causal variant. In such genomic contexts, Bayesian colocalization cannot fully separate the effects of tightly linked variants from large structural variations like the CFHR3/CFHR1 deletion [7], which exerts a powerful protective effect by removing the competitive antagonism of CFHR1. Functionally, whether this protective haplotype operates by deleting the competitive antagonist or by tagging increased expression of CFH, the downstream biological consequence converges on the unhindered, CFH-mediated regulation of the alternative pathway.

A biological paradox emerges when comparing our systemic genetic baseline with the local pathological microenvironment. In independent clinical cohorts, we observed that intra-renal CFH mRNA was upregulated during active disease, strongly correlating with worsened eGFR and heavy proteinuria. We initially hypothesized that this reflects a transcriptional-translational disconnect driven by massive complement consumption and urinary loss. During acute nephritic flares, the alternative pathway is heavily engaged, leading to the rapid consumption of CFH in the subendothelial and mesangial space where galactose-deficient IgA1 (Gd-IgA1) immune complexes deposit. Furthermore, as the glomerular filtration barrier fails, circulating CFH (a 155 kDa glycoprotein) is lost via proteinuria, exacerbating local depletion [2]. This functional exhaustion may contribute to a severity-driven compensatory upregulation of CFH mRNA, a phenomenon consistently observed in *in vitro* models of IgAN following nephritogenic stimulation [18]. Our co-expression network analysis, which highlighted an enrichment of homeostatic and negative regulatory pathways, is consistent with this compensatory defense hypothesis rather than a maladaptive pro-inflammatory role.

Alternative biological mechanisms warrant consideration. Because CFH functions as a key regulator of the complement system, which is dynamically modulated during inflammatory and acute-phase responses, its localized upregulation could be driven by generalized pro-inflammatory cytokines like IFN-γ rather than targeted homeostatic compensation [19]. Furthermore, the transcriptomic surge observed in bulk and microdissected glomeruli might simply reflect shifts in cell-type composition. The infiltration of CFH-expressing immune cells, particularly macrophages, into inflamed compartments would elevate the regional CFH mRNA signal even in the absence of intrinsic upregulation by resident renal cells [20].

We also acknowledge a technical caveat regarding these legacy transcriptomic validations. The high sequence homology within the 1q32 cluster raises the possibility of cross-hybridization artifacts in microarray datasets (e.g. GSE93798). We cannot exclude the possibility that the observed CFH upregulation inadvertently captures transcripts of its structurally analogous paralogs, such as the pathogenic CFHR1. Importantly, the current transcriptomic findings are associative in nature and do not allow definitive inference regarding the underlying biological mechanism. While our network analysis aligns with a homeostatic model, future studies using single-cell spatial transcriptomics and *in vivo* functional models are essential to distinguish compensatory CFH defense from inflammation-driven cellular shifts and paralog confounders.

Therapeutically, these insights provide a genomic rationale for the current landscape of complement-directed drug development. Advanced clinical agents for IgAN, such as iptacopan (Factor B inhibitor) and vemircopan (Factor D inhibitor), function by suppressing the enzymatic engine of the AP [19]. Genetic evidence supporting a protective role of CFH provides indirect support for the therapeutic rationale of these upstream inhibitors. Our findings also raise a hypothesis-generating translational possibility: recombinant CFH administration (e.g. GEM103). Our PheWAS data revealed that genetically predicted CFH enhancement is associated with a decreased risk of AMD, suggesting the potential for dual clinical benefit associated with modulation of this pathway.

However, direct CFH supplementation represents a hypothesis-generating observation rather than a clinically actionable conclusion, requiring rigorous validation in preclinical *in vivo* models. While our PheWAS screening indicates an absence of broad chronic pleiotropic diseases, it cannot capture acute pharmacological toxicities. Systemic AP inhibition or alteration is associated with an increased susceptibility to encapsulated bacterial infections, most notably *Neisseria meningitidis* [21]. Thus, these genetic findings support the theoretical rationale for CFH modulation but do not circumvent the necessity for rigorous preclinical toxicological evaluations and strict vaccination protocols in clinical implementation.

An epidemiological limitation warrants consideration. To mitigate population stratification, our MR and colocalization analyses were confined to cohorts of European ancestry. Given the epidemiological disparities where IgAN imposes a higher incidence and more rapid progression to end-stage renal disease in East Asian populations [22], the generalizability of these target effects requires validation in trans-ethnic or East Asian-specific cohorts. Additionally, we acknowledge key methodological constraints inherent to computational genetic epidemiology. Our eQTL instruments were derived from whole blood, which may underrepresent kidney-specific transcriptional nuances despite serving as a proxy for systemic regulation [14]. Furthermore, our reliance on circulating pQTLs and the absence of wet-lab functional validation necessitate cautious interpretation.

In conclusion, this multi-omics genetic triangulation provides evidence beyond observational correlations to support CFH as a genetically supported protective target in IgAN. By independently resolving the CFH-CFHR1 locus complexity and hypothesizing a severity-driven compensatory model for local transcription, this study provides a genomic framework to inform future investigation of alternative pathway regulators as potential precision therapeutics for IgA nephropathy.

## Supplementary Material

sfag176_Supplemental_File

## Data Availability

The datasets analysed during the current study are available in the following public repositories: UK Biobank Pharma Proteomics Project (UKB-PPP), deCODE Genetics, eQTLGen Consortium, Human Protein Atlas (HPA), CZ CELLxGENE Discover platform, Gene Expression Omnibus (GSE93798), and the MRC IEU OpenGWAS database. All data generated or analyzed during this study are included in this published article and its supplementary information files.
