# Supplementary material for: Multi-omics triangulation identifies complement factor H as a genetically supported protective factor in IgA nephropathy
Source: Clin Kidney J. 2026 May 29;19(7):sfag176. doi: 10.1093/ckj/sfag176 (PMC13320233; doi:10.1093/ckj/sfag176)
Supplement: sfag176_Supplemental_File [file sfag176_supplemental_file.docx]

**Supplementary Table S1. Full results of the forward mendelian randomization (MR) analysis evaluating the causal effects of circulating candidate proteins on IgA nephropathy risk.**

| **Target Protein** | **Method** | **No. of SNPs** | **OR (95% CI)** | **P-value** |
| --- | --- | --- | --- | --- |
| APRIL | Inverse variance weighted | 39 | 1.231 (1.088-1.393) | < 0.001 |
|  | Weighted median | 39 | 1.194 (1.037-1.374) | 0.014 |
|  | MR Egger | 39 | 1.275 (1.034-1.573) | 0.029 |
|  | Simple mode | 39 | 1.162 (0.914-1.476) | 0.227 |
|  | Weighted mode | 39 | 1.230 (0.628-2.410) | 0.549 |
| C3 | Inverse variance weighted | 4 | 1.522 (0.829-2.794) | 0.176 |
|  | Weighted median | 4 | 1.651 (1.038-2.625) | 0.034 |
|  | MR Egger | 4 | 1.864 (0.531-6.544) | 0.434 |
|  | Simple mode | 4 | 2.181 (0.494-9.626) | 0.379 |
|  | Weighted mode | 4 | 1.604 (0.297-8.653) | 0.621 |
| CFH | Inverse variance weighted | 23 | 0.747 (0.628-0.889) | < 0.001 |
|  | Weighted median | 23 | 0.742 (0.580-0.950) | 0.018 |
|  | MR Egger | 23 | 0.677 (0.477-0.961) | 0.041 |
|  | Simple mode | 23 | 0.577 (0.367-0.908) | 0.026 |
|  | Weighted mode | 23 | 0.746 (0.183-3.031) | 0.686 |
| CFHR1 | Inverse variance weighted | 22 | 1.436 (1.280-1.609) | < 0.001 |
|  | Weighted median | 22 | 1.522 (1.281-1.808) | < 0.001 |
|  | MR Egger | 22 | 1.448 (1.131-1.855) | 0.008 |
|  | Simple mode | 22 | 1.472 (1.129-1.919) | 0.009 |
|  | Weighted mode | 22 | 1.556 (0.969-2.498) | 0.082 |

**Supplementary Table S2. Strength of genetic instruments (F-statistics).**

| **Exposure** | **No. of SNPs** | **Mean F-statistics** | **Minimum F-statistics** |
| --- | --- | --- | --- |
| CFH | 23 | 264.072 | 22.745 |
| CFHR1 | 22 | 505.727 | 22.245 |
| APRIL | 39 | 165.310 | 20.147 |
| BAFF | 1 | 16.948 | 16.948 |
| C3 | 4 | 89.819 | 22.318 |

**Supplementary Table S3. Assessment of heterogeneity and horizontal pleiotropy.**

| **Exposure** | **Method** | **Cochran’s Q** | **P- heterogeneity** | **Egger intercept** | **P-intercept** |
| --- | --- | --- | --- | --- | --- |
| CFH | IVW | 33.838 | 0.051 | 0.018 | 0.530 |
| CFHR1 | IVW | 26.032 | 0.205 | -0.003 | 0.938 |
| APRIL | IVW | 40.515 | 0.278 | -0.004 | 0.732 |
| BAFF | IVW | 32.868 | 0.781 | 0.025 | 0.022 |
| C3 | IVW | 5.984 | 0.112 | -0.022 | 0.737 |

**Supplementary Table S4. Bidirectional mendelian randomization (MR) evaluating the causal effect of genetic liability to IgA nephropathy on circulating target protein levels.**

| **Outcome** | **No. of SNPs** | **Method** | **Beta (95% CI)** | **P-value** |
| --- | --- | --- | --- | --- |
| CFH | 25 | IVW | -0.154 (-0.364, 0.057) | 0.152 |
|  |  | MR Egger | -0.770 (-1.556, 0.016) | 0.067 |
|  |  | Weighted median | -0.008 (-0.040, 0.024) | 0.635 |
| CFHR1 | 26 | IVW | 0.416 (-0.029, 0.861) | 0.067 |
|  |  | MR Egger | 1.759 (0.135, 3.382) | 0.044 |
|  |  | Weighted median | 0.016 (-0.017, 0.049) | 0.338 |
| C3 | 25 | IVW | -0.006 (-0.035, 0.024) | 0.699 |
|  |  | MR Egger | -0.027 (-0.147, 0.093) | 0.665 |
|  |  | Weighted median | 0.001 (-0.030, 0.031) | 0.974 |
| APRIL | 25 | IVW | 0.044 (-0.148, 0.237) | 0.651 |
|  |  | MR Egger | -0.236 (-0.957, 0.485) | 0.528 |
|  |  | Weighted median | -0.046 (-0.075, -0.017) | 0.002 |
| BAFF | 25 | IVW | -0.017 (-0.057, 0.023) | 0.404 |
|  |  | MR Egger | 0.153 (0.008, 0.297) | 0.050 |
|  |  | Weighted median | 0.001 (-0.030, 0.031) | 0.968 |

**Supplementary Table S5. Transcriptome-wide Mendelian randomization (MR) evaluating the causal effect of CFH mRNA expression on IgA nephropathy risk.**

| **Exposure** | **Outcome** | **Analytical Method** | **No. of SNPs** | **OR (95% CI)** | **P-value** |
| --- | --- | --- | --- | --- | --- |
| CFH mRNA | IgA nephropathy | Weighted median | 3 | 0.718 (0.539-0.955) | 0.023 |
|  |  | IVW | 3 | 0.780 (0.246-2.478) | 0.673 |
|  |  | MR Egger | 3 | 0.042 (0.007-0.257) | 0.181 |

**Supplementary Table S6. Sensitivity analyses for the transcriptome-wide Mendelian randomization (MR) evaluating CFH mRNA expression.**

| **Sensitivity Test** | **Target** | **Method** | **Statistic** | **P-value** |
| --- | --- | --- | --- | --- |
| Heterogeneity | CFH mRNA | IVW (Cochran's Q) | Q = 43.68 | < 0.001 |
|  |  | MR-Egger (Cochran's Q) | Q = 3.72 | 0.054 |
| Horizontal Pleiotropy | CFH mRNA | MR-Egger Intercept | Intercept = 0.350 (SE = 0.107) | 0.189 |

**Supplementary Table S7. Multivariable mendelian randomization (MVMR) analysis mutually adjusting for CFH and CFHR1 on IgA nephropathy risk.**

| **Target Protein** | **Analytical Method** | **Beta (95% CI)** | **SE** | **P-value** |
| --- | --- | --- | --- | --- |
| CFH (Adjusted for CFHR1) | MVMR-IVW | -0.403 (-1.861, 1.054) | 0.744 | 0.625 |
| CFHR1 (Adjusted for CFH) | MVMR-IVW | 0.198 (-0.529, 0.926) | 0.371 | 0.630 |
